# Community Health Workers' Experiences in Strengthening the Uptake of Childhood Immunization and Malaria Prevention Services in Urban Sierra Leone

**DOI:** 10.3389/fpubh.2021.767200

**Published:** 2021-12-02

**Authors:** Atsuyoshi Ishizumi, Roberta Sutton, Anthony Mansaray, Lauren Parmley, Oliver Eleeza, Shibani Kulkarni, Tom Sesay, Laura Conklin, Aaron S. Wallace, Adewale Akinjeji, Mame Toure, Maria Lahuerta, Mohamed F. Jalloh

**Affiliations:** ^1^Immunization Systems Branch, Global Immunization Division, U.S. Centers for Disease Control and Prevention, Atlanta, GA, United States; ^2^ICAP at Columbia University, Mailman School of Public Health, New York, NY, United States; ^3^ICAP at Columbia University, Mailman School of Public Health, Freetown, Sierra Leone; ^4^Child Health and Immunization Program, Ministry of Health and Sanitation, Freetown, Sierra Leone; ^5^Department of Epidemiology, Mailman School of Public Health, New York, NY, United States

**Keywords:** community health worker (CHW), Sierra Leone, immunization, malaria, urban slum

## Abstract

**Introduction:** Community health workers (CHWs) play an integral role in Sierra Leone's health systems strengthening efforts. Our goal was to understand CHWs' experiences of providing immunization and malaria prevention services in urban settings and explore opportunities to optimize their contributions to these services.

**Methods:** In 2018, we conducted an exploratory qualitative assessment in the Western Area Urban district, which covers most of the capital city of Freetown. We purposively selected diverse health facilities (i.e., type, ownership, setting) and recruited CHWs through their supervisors. We conducted eight focus group discussions (FGD) with CHWs, which were audio-recorded. The topics explored included participants' background, responsibilities and priorities of urban CHWs, sources of motivation at work, barriers to CHWs' immunization and malaria prevention activities, and strategies used to address these barriers. The local research team transcribed and translated FGDs into English; then we used qualitative content analysis to identify themes.

**Results:** Four themes emerged from the qualitative content analysis: (1) pride, compassion, recognition, and personal benefits are important motivating factors to keep working as CHWs; (2) diverse health responsibilities and competing priorities result in overburdening of CHWs; (3) health system- and community-level barriers negatively affect CHWs' activities and motivation; (4) CHWs use context-specific strategies to address challenges in their work but require further support.

**Conclusion:** Focused support for CHWs is needed to optimize their contributions to immunization and malaria prevention activities. Such interventions should be coupled with systems-level efforts to address the structural barriers that negatively affect CHWs' overall work and motivation, such as the shortage of work supplies and the lack of promised financial support.

## Introduction

Although Sierra Leone continues to have one of the highest mortality rates among infants and children under 5 years of age (under-five) globally, some progress has been made. Infant mortality rate and under-five mortality rate declined from 190 to 80 per 1,000 live births and from 333 to 109 per 1,000 live births, respectively, between 1970 and 2019 (1). However, vaccine-preventable diseases (VPDs) such as measles and other preventable diseases such as endemic malaria pose serious challenges to the country's fragile health system while it continues to recover from 2014 to 2016 Ebola epidemic (2, 3). Maternal and child health (MCH) services, including immunization and malaria prevention services, were severely disrupted during the epidemic (4) due in part to bi-directional fear and stigmatization associated with Ebola among healthcare workers and patients, as well as resource constraints in the health system (5).

The Ebola epidemic's impact on Sierra Leone's VPD prevention activities is evidenced by an increase in the incidence of confirmed measles, which remained higher than pre-epidemic levels through 2017 (2). In 2019, 75% of the children aged 12–23 months in the country received the first dose of the measles-containing vaccine (MCV1) and 78% had received all three doses of the diphtheria-tetanus-pertussis (DTP) vaccine (6)—below the World Health Organization's goal of 95% MCV1 and 90% DTP vaccine coverage in the Africa region (7, 8). Globally, immunization programs in urban areas, including slums, face unique challenges. For instance, frequent population movement can impede the immunization program's ability to identify target populations and defaulters—a recurring challenge in urban settings in low- and middle-income countries (LMICs) (9). Estimates from an urban district in Sierra Leone indicate suboptimal vaccination coverage, with reported MCV1 coverage of 77% as of 2019 (8).

In addition to its VPD burden, Sierra Leone is among the 17 countries that account for 80% of all malaria deaths, globally (10). In 2016, the malaria prevalence among under-five children was 40% in the country (11). Recognizing the high burden of malaria in infants, Sierra Leone became the first country to nationally implement intermittent preventive treatment of malaria in infants (IPTi) via its existing childhood immunization program (12).

Recent estimates of the vaccination coverage and malaria incidence in Sierra Leone show that both have been improving (13, 14), which is a testament to the intensified post-Ebola health systems strengthening efforts. As part of these efforts, community health workers (CHWs) play an integral role especially given the country's ongoing health system challenges, such as the shortage of human resources (15). CHWs comprise a diverse category of laypersons who commonly work outside of the fixed health facilities and have some formal, yet limited, training related to the specific tasks they perform (16). The effectiveness of CHW programs in improving an array of health outcomes has been documented elsewhere, including those related to immunization and malaria prevention and treatment (16, 17). In Sierra Leone, the national CHW programme was formally launched in 2012 (18). Although the estimated 15,000 CHWs in Sierra Leone are not considered part of the National Civil Service (19, 20), they act as the first contact point for health problems at the community level (21). In addition to community sensitization and health promotion, they are expected to perform duties such as rapid diagnostic tests for malaria and referral of defaulted children to health facilities for catchup vaccination (21–23). Changes in CHWs' responsibilities during the Ebola epidemic have been documented (e.g., suspension of activities that require physical contact) (21), but there is limited literature on their experiences in the post-Ebola context especially as they relate to immunization and malaria prevention activities in urban areas.

As Sierra Leone sought to rebuild trust and confidence in child health services after the Ebola epidemic ended, we aimed to gain a qualitative understanding of the role and experiences of CHWs in promoting immunization and malaria prevention services in an urban context. The assessment was carried out as part of larger mixed-methods assessment for understanding the range of barriers and facilitators that may influence the delivery of immunization and malaria prevention services in urban areas in Sierra Leone, including slums.

## Methods

In 2018, we conducted an exploratory qualitative assessment with CHWs in an urban district in Sierra Leone to (1) characterize the nature of their work and their attitudes toward it, (2) identify barriers to their work and strategies currently used to address them, and (3) explore opportunities that optimize their contributions to community health, with a focus on improving vaccination demand and malaria prevention. The methods of the assessment have been described in accordance with the Consolidated Criteria for Reporting Qualitative Research (24), a widely accepted framework for reporting qualitative methods such as focus group discussions (FGD).

### Study Setting

We conducted this qualitative assessment in Sierra Leone's Western Area Urban (WAU) district. With an estimated population of 1,055,964, it is the most populous district in the country and covers most of the capital city of Freetown (25). During Sierra Leone's civil conflict between 1991 and 2002, the district experienced rapid urbanization, and many unplanned slums were created (26). It is also a destination for people from rural communities, seeking economic opportunities; therefore, it has a transient population residing in informal settlements (27, 28). The district was severely affected by the Ebola epidemic, reporting a total of 3,142 cases during the epidemic period (27).

Sierra Leone has ~1,300 health facilities across 16 districts. All health facilities in Sierra Leone are assigned a set of geographic catchment communities that they serve. A total of 10 CHWs are assigned to each health facility to support the promotion and utilization of essential primary care services including malaria prevention and routine childhood immunization. Each set of 10 CHWs are overseen by a supervisory CHW who is also the CHW liaison to the assigned health facility.

### Sampling and Recruitment

We purposively selected eight health facilities in the WAU district to ensure diversity in facility type (community health center, maternal child health post, and hospital), ownership (private, public, non-governmental organization), zones (west, central, east), and community settings (slum or non-slum) (Table 1). All 10 CHWs assigned to each of the eight facilities were invited to participate in the FGDs if they were available and willing. Their recruitment was coordinated through their supervisors. Because the supervisors of the CHWs assisted in the recruitment and because of the possibility of socially desirable response bias, they were excluded from participating in the assessment. We did not collect demographic information of the individual CHWs because our unit of analysis was at the group level, namely the collective experience of urban CHWs as a functional unit. It was also deemed inappropriate to collect personal information of the CHWs due to complications involved in protecting the identity of individuals in small known groups.

**Table 1:** Characteristics of the focus group discussions with community health workers (CHWs), qualitative assessment, Western Area Urban, Sierra Leone, 2018.

**Descriptor**	**Characteristic**	**Number of FGDs**
**Community**	**Community setting**	
	Urban slum communities	4
	Urban non-slum communities	4
	**Geographic zone**	
	West	2
	Central	3
	East	3
**Health facility**	**Type of health facility**	
	Hospital	2
	Community health center Maternal child health post	2 4
	**Ownership of health facility**	
	Private	3
	Public (government owned)	4
	Non-governmental organization	1

### Data Collection

First, we piloted the moderator guide with one FGD to refine the questions and probes and excluded its transcript from the analysis. Then, the moderators used the revised guide to facilitate the eight FGDs, and each FGD consisted of 6–8 participants. The guide was orally translated into Krio, the most widely spoken language in the WAU district that is predominantly oral. The topics explored included participants' background, responsibilities and priorities of urban CHWs, sources of motivation at work, barriers to CHWs' immunization and malaria prevention activities, and strategies used to address these barriers. Data collection took place in August–September 2018.

The staff facilitating the FGDs were trained in qualitative research methods, had experience conducting FGDs in Sierra Leone and could speak Krio. The FGDs were held in pre-identified community-based settings around 8 health facilities included in the assessment. The data collection teams liaised with the CHWs' peer-supervisors to ensure that the location was quiet and conducive for the discussion. One facilitator and one note-taker participated in each FGD. We audio-recorded the FGDs with the permission of participants;. The FGDs lasted for approximately 90 min. The data collection team held a debriefing session after each FGD to immediately document their observations, make note of the key points raised by CHWs, and identify ways to improve probing questions in the subsequent FGDs. The audio-recordings from the FGDs were transcribed and translated jointly by the same two team members that collected the data. The process usually took place in three steps: (1) they listened to short segments of the audio recording together, (2) one of them translated the audio segment from Krio to English, (3) the other team member either agreed with the translation or provided suggested edits that they then discussed and agreed upon. Whenever the two could not reach consensus during translation, then a locally hired supervisor with advanced proficiency in Krio was consulted to assist with resolving the translation issue. This process continued until each audio recording was fully translated and transcribed.

### Data Analysis

Two analysts under the supervision of a senior analyst who has extensive experience conducting qualitative research in Sierra Leone analyzed the data, using qualitative content analysis. First, the two analysts read the transcripts and summarized textual excerpts into condensed meaning units (29). Second, each analyst independently coded the meaning units and compared their lists of codes, addressed discrepancies through discussion, and iteratively created a unified set of inductive codes. Third, they grouped the codes into mutually exclusive categories that described various underlying concepts. Finally, they identified the themes that cut across multiple categories of codes through an interpretative, iterative process. To assess data saturation, we used an approach proposed by Malterud, wherein we examined the extent to which our sample provided adequate information power for the requisite analysis to address the aims of the assessment (30). We organized the codes, categories, and themes, using Microsoft Word and Excel.

### Ethical Considerations

All participants provided a written, signed, or thumb-printed informed consent before joining the FGDs. The assessment was approved by the Columbia University Medical Center Institutional Review Board and Sierra Leone Ethics and Scientific Review Board. The assessment was determined to be non-research by the U.S. Centers for Disease Control and Prevention's Human Subjects Office. The participants received no compensation.

## Results

Table 1 summarizes the community- and facility-level characteristics of the FGDs. Tables 2.1–2.4 outlines the categorization of the meaning units into themes. Four themes related to different aspects of CHW experiences emerged from the FGDs. The themes describe the underlying motivation for CHWs' work, various challenges they experienced in their work, and the strategies they used to overcome recurring challenges.

**Table 2.1:** Codes and categories for Theme 1: “Pride, compassion, recognition, and personal benefits are important motivating factors for CHWs”, qualitative assessment, Western Area Urban, Sierra Leone, 2018.

**Code**	**Category**
Past related experience	Exclusive process of becoming CHW
Emphasis on being selected	
Receiving training before becoming CHW	
Wanting to improve general health	Desire to improve health
Wanting to improve MCH	
Wanting to improve malaria prevention	
Perceived Impact of CHW activities on community health	
Community wanting us to always be there	Positive recognition from community
Increased popularity among community members	
Increased respect among community members	
Positive interactions with community members	
Increased health knowledge and skills as a benefit	Personal benefits with CHW status
Better access to health facility as a benefit	
Official government status as a benefit	

*CHWs, community health workers*.

**Table 2.2:** Codes and categories for Theme 2: “Diverse health responsibilities and competing priorities result in overburdening of CHWs”, qualitative assessment, Western Area Urban, Sierra Leone, 2018.

**Code**	**Category**
MCH as a priority area	Top CHW priorities
Malaria prevention as a priority area	
Immunization as a priority area	
Hygiene as a priority area	
Older adults as a priority area	
Community outreach as a priority area	
Patient referral as a priority area	
Patient referral and escorting	General health activities
Community education on MCH (maternal and child health)	
Community education on general health	
Community education on WASH (water, sanitation, and hygiene)	
Other community outreach activities	
Engagement with other organizations	
Miscellaneous activities	
Community education on immunization	Immunization activities
Defaulter tracing	
Involvement in immunization campaigns	
Community education on malaria prevention	Malaria prevention activities
Performing malaria diagnostic tests	
Bed net distribution	
Administration of malaria treatment	
Involvement in other jobs	Time management
High workload and demand	

*CHWs, community health workers*.

**Table 2.3:** Codes and categories for Theme 3: “Health system and community-level barriers negatively affect CHWs' activities and motivation”, qualitative assessment, Western Area Urban, Sierra Leone, 2018.

**Code**	**Category**
Delay or lack of stipend disbursement	Lack of stipends
Sense of betrayal due to lack of stipend	
Community members not going to health facility	Challenges related to patient referral
Poor access to health facilities	
Lack of medical supplies	
Not adhering to CHW health advice	Challenges related to community education and outreach
Negative attitudes toward CHWs	
Lack of identification	
Shortage of materials	
Caregivers misplacing or not showing children's health cards	Challenges related to immunization activities
Reluctance or refusal among Fulas	
Seeking immunization from providers of choice	
Fear due to not following vaccine schedule	
Concerns about adverse events	
Hard-to-reach areas	
Stockout or lack of bed nets	Challenges related to malaria prevention
Lack of rapid test kits	
Lack of malaria drugs	
Community members refusing to use bed nets	
Community members complaining about bed nets or not using them properly	
Difficulty in CHW collaboration across communities	

*CHWs, community health workers*.

**Table 2.4:** Codes and categories for Theme 4: “CHWs use context-specific strategies to address challenges in their work but require further support”, qualitative assessment, Western Area Urban, Sierra Leone, 2018.

**Code**	**Category**
Interpersonal communication	Current strategies
Community engagement	
Help from senior health workers	
Suggestion for addressing structural challenges	Proposed strategies for improvement
Suggestion for improving community education	

*CHWs, community health workers*.

### Theme 1: Pride, Compassion, Recognition, and Personal Benefits Are Important Motivating Factors to Keep Working as CHWs

CHWs took great pride in their work and were highly motivated to improve community health. They particularly expressed that their work has a positive impact on the health of women and children—a responsibility that they took seriously. Most participants in our assessment had already been volunteering in a myriad of health areas before they were eventually selected or recruited to receive further training to become CHWs. They stressed the exclusivity of the journey to becoming a CHW, which strongly reflected their pride and sense of responsibility in their duties.

“I have worked as a CHW for 1 year because of the suffering of the people, especially pregnant women. We talk to them to attend the clinic. Sometimes they are not happy, but we encourage them… I am happy to join the CHW [program] because it has reduced disease cases.”—CHW, FGD 2, non-slum.

They emphasized that they have become “more popular” in their communities because of working as CHWs. Similarly, some mentioned that they have gained “more respect” from the community. Positive recognition sometimes translated into demonstrations of appreciation for CHWs through in-kind offerings and gestures from community members.

“A lot of people also know me now in the community. Like for instance, sometimes when I board a vehicle, I will not pay at all as someone in the vehicle who might have gained from advice I have given to community members will say ‘my sister, don't pay, I'll pay for you as you spoke to me the last time and I really saw the benefit of what you told me.”—CHW, FGD 5, non-slum.

CHWs acquired new skills and knowledge through their work, which helped them professionally as well as in their personal lives. For example, participants shared that they were able to care for sick family members because of what they had learned during their CHW engagements. Another perceived benefit among CHWs was the ability to gain access to healthcare facilities and build relationships with healthcare workers.

“Before now I've got no business at this health facility, but now I'm proud to say that, everyone in this facility knows me by name and by face. Now I can access the facility at any time and also help the nurses in doing their work.”—CHW, FGD 1, slum.

The first thematic area strongly pointed to CHWs' personal pride and dedication to community health that helped them stay motivated. They also gained certain benefits from their work, such as new skills and community recognition, which elevated their social standing and enhanced their ability to help people. Despite these motivating factors, CHWs sometimes appeared to be overburdened, which has been elaborated in the second thematic area.

### Theme 2: Diverse Health Responsibilities and Competing Priorities Result in Overburdening of CHWs

CHWs performed wide-ranging activities, including daily household visits in their designated communities, referral or escort to local healthcare facilities, and support for MCH activities in areas, such as community education and outreach. Their activities for malaria prevention included distributing bed nets, providing community education on bed net usage as well as water, sanitation, and hygiene (WASH) issues, performing rapid diagnostic tests, and distributing medications for treatment. Specific to immunization, CHWs actively promoted immunization via community education and reminders. They described playing an active role in defaulter tracing, which usually involved scanning of child health cards during household visits or conversations with defaulters to promote catch-up vaccination.

“We don't just ask the mothers whether their children have taken the immunization or not but rather we'll ask for their cards… If we discover a mother who has become defaulted, maybe the time stated on the card has passed and she has not taken the child for immunization, we'll not be angry with her but rather we'll try to know the [cause]. And with a soft voice, try to encourage her to take the child for the immunization. Sometimes we'll even go with them and make sure they go to the health facility.”—CHW, FGD 3, slum.

It was evident in the discussions that CHWs were responsible for a wide range of health promotion tasks that overburden them and require prioritization. Many considered MCH (e.g., supporting pregnant women and sick children) to be an important area of work. Some CHWs explicitly referred to childhood immunization when describing their priorities. Other priority areas mentioned included malaria prevention, hygiene, and household visits. CHWs recurrently expressed that some of their activities particularly demanded significant time commitment, including the community health education and outreach sessions. Some community members visited CHWs' homes to seek advice, which meant that they had to respond even when they were not on duty. Moreover, they often had to work late hours to help community members navigate health emergencies.

“Sometimes when I go out during the night, I use to pass the night if there's any case especially when it comes to the pregnant women or when it has to do with a pregnant [woman] who may have to be admitted to the hospital who might have got an attack during the night, in such a case I use to pass the night going up and down from the affected household to the hospital until there's a permanent solution.”—CHW, FGD 6, slum.

In addition to the long and extra hours CHWs volunteer, they had income-generating responsibilities to support themselves and their families. This meant that they had to balance multiple jobs, such as small retail businesses, petty trading, teaching, and hairdressing in addition to frequently being the primary caregiver of their own children. Overall, heavy workload and multiple work responsibilities were commonly noted as a part of being a CHW. Although CHWs did not explicitly tie their heavy workload to their performance, it is possible that their performance may be hindered by having an unrealistic workload under a volunteer scheme. This becomes more evident in the third thematic area where performance-related connections are made between workload and other systemic challenges.

### Theme 3: Health System and Community-Level Barriers Negatively Affect CHWs' Activities and Motivation

The lack of stipend disbursement was a major source of frustration and demotivation among CHWs. Although CHWs were initially told to expect financial support for their work, they expressed that they had not received stipends as promised, which was viewed as a form of “betrayal” by the CHW program. The lack of financial support was also mentioned as a reason for pursuing multiple income-generating activities outside of their CHW volunteer duties.

“They promised to give us incentives…it is just a lot of promises and nothing good has come out of those promises… What is more painful is that they'll just call us sometimes and pass information that [they] are going to give us the money…but that hasn't come to past till now. There were colleagues of ours who have been waiting for this money and have died without receiving it. Some have got different plans for that money… they were going to pay the school fee of their children.”—CHW, FGD 7, non-slum.

CHWs faced a series of challenges with patient referrals. Some community members were reportedly reluctant to go to health facilities because of the perceived financial costs of healthcare services. CHWs also cited access challenges, such as the refusal of services.

“I've [had] an incident in which two of the pregnant women that I referred to the health center were rejected. I filled a form for them and sent them to the hospital but when they showed up, they were rejected by the nurse. The nurses said, we should not be the ones to go and register people and refer them to the health center.”—CHW, FGD 1, slum.

A common challenge with community education and outreach was that some community members did not adhere to CHWs' health advice. Community members' distrust in CHWs, which sometimes stemmed from rumors about CHWs' involvement in financial fraud, was also reported. Community outreach was further complicated by the lack of work identification cards, which prevented public acknowledgment and evidence of CHWs' official status when carrying out their duties.

CHWs also encountered challenges that were specific to immunization activities. Sometimes defaulters were not easily identified because it was difficult to obtain child health cards from caregivers who were either uncooperative or had misplaced them. Though uncommon, CHWs encountered instances where caregivers refused recommended immunization services because they claimed to be receiving services from “private doctors”. Additionally, the Fula community, an ethnic group residing in the WAU district, was perceived by CHWs as unaccepting of immunization services for unspecified reasons.

“Our biggest problem for this immunization is the Fulas. They're very stubborn when it comes to issues of immunization, even if you explain to them the importance of the immunization, and they'll still try to give you an excuse so that they'll prevent their children from taking the immunization.”—CHW, FGD 4, slum.

CHWs stated that, based on misinformation, some caregivers were worried about adverse events following immunization. They purportedly believed that vaccination may have harmful effects on their children's short- and long-term health. For example, one CHW noted:

“[Defaulters] are saying, their children get malaria through the injection they give them, so they will not take their children to any hospital for any treatment that has to do with giving them an injection.”—CHW, FGD 8, non-slum.

CHWs encountered situations where community members refused to use bed nets or opted to use them for other livelihood purposes, such as agriculture. Common complaints CHWs received about bed nets included discomfort associated with heat generated while sleeping under bed nets. In addition, the lack of malaria test kits and medications was cited as a barrier that limited CHWs' contributions to malaria control.

In this third thematic area it was evident that CHWs' work involved navigating complex challenges resulting from community perceptions and health practices, as well as health systems issues beyond their control. As outlined in the fourth thematic area, however, CHWs were able to develop context-specific strategies to mitigate recurring challenges.

### Theme 4: CHWs Use Context-Specific Strategies to Address Challenges in Their Work but Require Further Support

CHWs used interpersonal communication techniques to address some of the challenges they encountered in their communities. They persistently communicated with uncooperative community members and made multiple household visits to engage them. Sharing their own positive experiences with vaccination was another strategy CHWs used to persuade some caregivers to accept vaccination.

“The woman was so desperate telling me not to think of giving her child the immunization so I allow her to express herself, then later I apply my own experience to make sure she knows the importance of the immunization. When I finished with my explanation, she came to realize the importance of the immunization and later told me to please inform her about the next immunization schedule.”—CHW, FGD 3, slum.

Community engagement was another way in which CHWs addressed community-level challenges. CHWs collaborated with members of the Fula community to conduct sensitization and communicate the importance of immunization. In other cases, they sought assistance from their supervisors when confronted with particularly challenging situations.

“We do identify that house that refuses vaccination with a symbol for the supervisor to know… to inform them about the refusal of immunization and the nurses do come and [those who were originally reluctant] will allow the nurses to administer it.”—CHW, FGD 2, non-slum.

CHWs also proposed potential strategies for improving their work. Most of their suggestions pertained to different types of support needed to address structural challenges in their work. Their suggestions, which were mostly directed at the government, included asking for increasing the supply of materials and medications, improving management and accessibility of healthcare facilities, creating a sanitary environment by providing communal trash cans, and securing financial support. They suggested that their work would be more effective if the government empowered them through these avenues, as one CHW noted:

“If we tell people to use veronica bucket [water container with a tap used for handwashing] and wash their hands then when they go and visit our households, we don't have anything like that, then it becomes ridiculous on our side. Therefore, [the] government shouldn't neglect our role as they have been doing but also capacitate us so the community will continue to listen to us and do what we tell them when it comes to immunization, malaria prevention, and many other things.”—CHW, FGD 7, non-slum.

## Discussion

Our qualitative assessment provides important insights into the experiences of CHWs working in an urban context in Sierra Leone, including slums. We found that CHWs in urban capital of Sierra Leone were overburdened and had insufficient support for overcoming the multitude of barriers they experienced in their work. These barriers negatively affected their motivation and abilities to effectively carry out immunization and malaria prevention activities. Nonetheless, CHWs valued the altruistic nature of their work and remained motivated to improve the effectiveness of their work, including employment of adaptive strategies in responding to the challenges encountered. Additional support from the health system is needed to enhance their contributions to immunization and malaria prevention activities. Critical barriers such as the shortage of supplies and the lack of financial support require systems-level interventions.

Motivating factors described by the CHWs in our assessment largely overlap with those from previous studies (31–35). A study carried out in Tanzania found that dedication to public service, desire for health knowledge, personal pride, and positive reception by community members served as sources of CHW motivation (32). Other studies have explored different interventions designed to enhance these motivations, such as supportive supervision to equip CHWs with new knowledge and skills (36) and participatory community activities to improve the community perception of CHWs (37). Factors such as the pride CHWs take in their work and satisfaction received from social recognition by their community should be maximized to motivate CHWs.

Previous research suggests that CHWs in LMICs tend to be inundated with many tasks (22, 33, 38), which reinforces the results of our assessment. In addition to multiple responsibilities, research from Sierra Leone, Liberia, and the Democratic Republic of Congo suggests that CHWs sometimes take on second jobs because financial incentives they receive are limited, and this may result in CHWs being not fully committed to their healthcare duties (31, 39). Unfulfilled stipend disbursements demotivated CHWs in our assessment, which is also a persistent challenge in other settings (33, 40, 41). Our assessment adds to the evidence that CHW financing needs to be prioritized to strengthen and maintain their essential role in community health engagement. This issue requires larger systems-level attention of developing different funding mechanisms for CHW financing and assessing needs for CHW scope of work that aligns with country priorities in different health areas (42, 43).

The shortage of medical supplies and other work equipment for CHWs has been well-documented (17, 31, 44). To avoid disruptions to CHWs' performance and confidence in the health system, system-level measures should be considered to address the barrier in a sustainable fashion. In the context of COVID-19, it is particularly important to ensure CHWs have access to essential supplies because they are expected to continue playing a critical role in the ongoing pandemic response (45).

A household survey conducted in the WAU district echoed the findings from our assessment regarding the fear of side effects and missing child health cards as barriers to immunization uptake (8). Another study in the WAU district found little evidence of CHWs' involvement in structured defaulter tracing, such as the use of defaulter lists generated from an immunization registry (23). Although CHWs in our assessment indicated that they played a role in defaulter tracing, it is unclear how structured their involvement was. CHWs in our assessment also reported the challenges relating to vaccine acceptance by the Fula ethnic group. This could be explained by religious convictions regarding vaccination or other social and behavioral factors as evidenced by studies involving Fula populations in other settings (46–48). More research is needed to understand the reasons for vaccine refusal among the Fula ethnic group, including traditional or religious beliefs (49), and how best to support CHWs in addressing this challenge—especially because Fula populations also reside in other parts of West Africa. Given the high level of trust CHWs receive as messengers of health information (8, 50), interpersonal communication could be considered as a strategy to address vaccine acceptance issues. Future research can explore in more depth the interpersonal communication techniques currently used by CHWs, how effective they are, and whether there are opportunities to further build capacity in this area.

Taken together, our findings point to the overarching need to consider interventions targeted at the specific barriers that CHWs face in their immunization and malaria prevention activities. These interventions should be coupled with systems-level efforts to define an achievable set of duties for CHWs to allow them to perform realistic workload as volunteers. Otherwise, to accommodate an expansive workload, Sierra Leone may have to formally integrate CHWs into the paid cadre of the health workforce, which would realistically enable them to meet expanded responsibilities that require full-time commitments and help ensure their performance quality. Such integration, however, may require careful and strategic allocation of financial resources for health services. Non-monetary incentives, which have previously been shown to be effective motivating factors, could also be considered (51). Beyond cost implications, integrating CHWs into the formal health system requires effective supportive supervision, continuous on-the-job training, and greater collaborations with healthcare workers (52).

Our assessment has several limitations. The involvement of the CHWs' supervisors in the recruitment process could have potentially led to CWHs providing socially desirable responses. To address the potential social desirability bias, we excluded the supervisors from participating in the FGDs so that CHWs could candidly share their opinions. Moreover, we explicitly explained to participants the voluntary basis of the assessment while obtaining informed consent. Another limitation is the variability of probing among data collection teams. A few potentially important issues were not adequately followed-up to gather more information in some FGDs. For instance, in one of the FGDs, it became evident that there were potential tensions between CHWs and the paid cadre of formal healthcare workers, which resulted in healthcare workers refusing to accept some referrals made by CHWs.

## Conclusion

Our qualitative assessment revealed the important role of CHWs in an urban district in Sierra Leone while the country was rebuilding public trust in the health system after the 2014–2016 Ebola epidemic. Although our assessment focused on a single urban district, it would be critical to ensure the availability of essential supplies and the promised stipends throughout the country. Moreover, the potential tensions between CHWs and the paid healthcare workforce may require further investigation in urban and rural settings. Given the severe shortage of a paid healthcare workforce in Sierra Leone, CHWs will continue to fulfill an important role in the health system. Focused support to facilitate CHWs' work for immunization and malaria services should therefore be considered, while also addressing structural barriers that negatively affect their overall work and motivation.

## Data Availability Statement

The datasets presented in this article are not readily available because of reasons related to data confidentiality and participant privacy. Requests to access the datasets should be directed to Mohamed F. Jalloh, yum8@cdc.gov.

## Author Contributions

MJ oversaw all aspects of the assessment. RS, AM, LP, OE, SK, TS, LC, AW, AA, MT, and ML contributed to the assessment's conceptualization, data acquisition, and/or analysis. AI led the analysis and manuscript development. All authors reviewed and approved the manuscript.

## Funding

This assessment was funded by the US Centers for Disease Control and Prevention via a cooperative agreement with ICAP-Columbia University (award # NU19GH001581-03-09). AI and SK were supported by the Oak Ridge Institute for Science and Education (ORISE) through their appointment to the Research Participation Program at the US Centers for Disease Control and Prevention.

## Conflict of Interest

The authors declare that the research was conducted in the absence of any commercial or financial relationships that could be construed as a potential conflict of interest.

## Publisher's Note

All claims expressed in this article are solely those of the authors and do not necessarily represent those of their affiliated organizations, or those of the publisher, the editors and the reviewers. Any product that may be evaluated in this article, or claim that may be made by its manufacturer, is not guaranteed or endorsed by the publisher.
